# Mitochondrial genetic haplogroups and cardiovascular diseases: Data from the Osteoarthritis Initiative

**DOI:** 10.1371/journal.pone.0213656

**Published:** 2019-03-28

**Authors:** Nicola Veronese, Brendon Stubbs, Ai Koyanagi, Alberto Vaona, Jacopo Demurtas, Patricia Schofield, Stefania Maggi

**Affiliations:** 1 National Research Council, Neuroscience Institute, Aging Branch, Padova, National Institute of Gastroenterology “S. De Bellis” Research Hospital, Castellana Grotte (Ba), Italy; 2 South London and Maudsley NHS Foundation Trust, Denmark Hill, London, United Kingdom; 3 Research and Development Unit, Parc Sanitari Sant Joan de Déu, Fundació Sant Joan de Déu, CIBERSAM, Sant Boi de Llobregat, Barcelona, Spain; 4 Primary Care Department, Azienda ULSS20 Verona, Verona, Italy; 5 Primary Care Department, Azienda USL Toscana Sud Est, Grosseto, Italy; 6 Faculty of Health, Social Care and Education, Anglia Ruskin University, Chelmsford, United Kingdom; Harvard Medical School, UNITED STATES

## Abstract

**Background:**

Some case-control studies reported that mitochondrial haplogroups could be associated with the onset of cardiovascular diseases (CVD), but the literature regarding this topic is limited. We aimed to investigate whether any mitochondrial haplogroup carried a higher or lower risk of CVD in a large cohort of North American people affected by knee osteoarthritis or at high risk for this condition.

**Materials and methods:**

A longitudinal cohort study including individuals from the Osteoarthritis Initiative was done. Haplogroups were assigned through a combination of sequencing and PCR-RFLP techniques. All the mitochondrial haplogroups have been named following this nomenclature: HV, JT, UK, IWX, and superHV/others. The strength of the association between mitochondrial haplogroups and incident CVD was evaluated through a Cox’s regression analysis, adjusted for potential confounders, and reported as hazard ratios (HRs) with their 95% confidence intervals (CIs).

**Results:**

Overall, 3,288 Caucasian participants (56.8% women) with a mean age of 61.3±9.2 years without CVD at baseline were included. During a median follow-up of 8 years, 322 individuals (= 9.8% of baseline population) developed a CVD. After adjusting for 11 potential confounders at baseline and taking those with the HV haplotype as reference (the most frequent), those with JT carried a significant lower risk of CVD (HR = 0.75; 95%CI: 0.54–0.96; p = 0.03). Participants with the J haplogroup had the lowest risk of CVD (HR = 0.71; 95%CI: 0.46–0.95; p = 0.02).

**Conclusions:**

The presence of JT haplogroups (particularly J) may be associated with a reduced risk of CVD. However, this result was not based on a high level of statistical significance. Thus, future research with larger sample size is needed to assess whether our results can be corroborated.

## Introduction

The human mitochondrial genome is a circular set of 16,569 base pairs encoding 37 genes, which are translated into 13 proteins involved in the electron transfer chain, a process essential for cellular function and survival [1]. Mitochondrial DNA (mtDNA) often undergoes mutations, though at a much higher rate than nuclear DNA replication rate, while DNA repair mechanisms are less efficient [1, 2]. In addition, the evolution of mtDNA occurs at a more rapid pace compared to the average nuclear DNA, and thus mutations have accumulated sequentially along radiating maternal lineages [3]. Mismatch can lead to single nucleotide polymorphisms (SNPs), and clusters of these specific SNPs in the mitochondrial genome define mitochondrial haplogroups. Not only germline mutations of the nuclear DNA can be on the basis of the genetic susceptibility to different diseases including cancer [4, 5], but also the biology of mtDNA may explain in part the genetic predisposition to certain pathological processes: mutations in mtDNA, in fact, may influence propensity of subjects to several medical conditions [6–8]. Moreover, inherited mutations of mtDNA lead to several diseases in children that mainly affect central nervous system, muscles and the heart [9].

The literature regarding mtDNA and cardiovascular disease (CVD) is increasing, but still limited to a small number of studies, although there is emerging evidence that altered mitochondrial metabolism might play a role in the development of CVD [10–13]. In two interesting reviews, the authors suggested that mitochondria are directly involved in the caloric conversion to energy, thermogenic output, and oxidant production, and all these factors are reported to be important for cardiovascular dysfunction [11, 13]. Moreover, mitochondrial-nuclear relationships were established millions of years ago, but the factors that could interfere with this genetic predisposition are largely unknown [11, 13]. In this sense, in animal models, it was reported that to change mtDNA background significantly changes the susceptibility to the pathological stress of cardiac volume overload, further reinforcing a role for mtDNA alterations in cardiac function [12].

In human beings, in one case-control study involving 406 participants with early myocardial infarction and 183 healthy controls, the authors found that the prevalence of two haplotypes (H1 and U5) was higher in those with myocardial infarction versus controls [14]. On the contrary, another case-control study, including 358 participants with ischemic cardiomyopathy compared to 423 healthy controls, reported that haplogroups H and J are associated with a significant higher and lower presence of ischemic cardiomyopathy, respectively [15]. The haplogroup J seems to be a protective factor also for the development of hypertrophic cardiomyopathy, as shown by another case-control study [16]. Finally, in a study involving 487 subjects with coronary artery disease (CAD), haplogroup T was significantly more prevalent among patients with CAD than among control subjects and, in diabetic patients, the presence of diabetic retinopathy was also significantly associated with a higher prevalence of haplogroup T than controls [17]. However, a longitudinal study conducted among 9,254 Danish participants followed-up for 25 years did not find any association between mitochondrial haplotypes and the incidence of CVD [18], indicating that more research is needed to understand whether mitochondrial haplotypes are associated with CVD.

Given this background, we aimed to investigate whether any mitochondrial haplogroup carried a significantly higher or lower risk of CVD in a large prospective cohort of North American people affected by knee osteoarthritis or at high risk for this condition since OA can increase per se the risk of CVD.[19].

## Patients and methods

### Data source and subjects

All subjects were recruited as part of the Osteoarthritis Initiative (OAI) study, freely available at http://www.oai.ucsf.edu. Specific datasets for this work are: baseline and screening evaluations (November 2008) (V00) and those evaluating the participants until the last evaluation available (96 months; V10). Patients at high risk of knee osteoarthritis or having knee osteoarthritis were recruited from four clinical sites in the USA (Baltimore, MD; Pittsburgh, PA; Pawtucket, RI; and Columbus, OH) between February 2004 and May 2006.

All participants provided written informed consent. The OAI study protocol was approved by the institutional review board of the OAI Coordinating Center, University of California at San Francisco.

For this specific research, we have conducted the data elaboration and wrote the manuscript, whilst the data collection and the administrative tasks were conducted by the OAI team.

### Exposure

The haplogroup assignment was performed in agreement with other studies [20], i.e. a combination of sequencing and PCR-RFLP techniques. The sequencing technique consisted in the multiplex assignment of the main 6 SNPs contributing to the generation of the most frequent Caucasian haplogroups [21] (H, V, super HV, U, K, T, J), following the single base extension (SBE) assay as reported in S1 Table.

All the mitochondrial haplogroups have been consequently named in accordance with this nomenclature suggested by the OAI (http://www.oai.ucsf.edu/): H, U, K, J, T, V, SuperHV, I, W, X or others. After that, we clustered these haplogroups following the classification suggested by MITOMAP and followed by another study assessing mitochondrial haplogroups and CVD [15, 22] into HV, JT, UK, and IWX. The analyzed haplogroups, in fact, share a common ancestor and several SNPs have been conserved during evolution. [15] The remaining two groups (superHV/others) were clustered together. The superHV group used as polymorphism the following restriction fragment (m.14766C.T) and as restriction enzyme -*Mse*I [23].

### Outcomes

The main outcome of interest was the onset of CVD during the follow-up period. The presence of CVD was recorded through self-reported information. We defined the development of CVD as the presence of heart attack, heart failure, unclog or bypass arteries in legs, and stroke, cerebrovascular accident, blood clot in brain, or transient ischemic attack. The presence of CVD in the OAI was recorded, other than baseline, during the V3 (24 months), V6 (48 months) and V10 (96 months) [24].

### Covariates

We identified a number of potential confounders in the relationship between mitochondrial haplogroups and CVD. These included: (1) physical activity evaluated through the Physical Activity Scale for the Elderly [25]. This scale covers twelve different activities (e.g. walking, sports, and housework) scoring from 0 to 400 and more; (2) smoking habits as “previous/current” vs. never; (3) educational level categorized as “degree” vs. others; (4) yearly income as < vs. ≥ 50,000 $ or missing data; (5) co-morbidities assessed through the modified Charlson comorbidity score, with higher scores indicating an increased severity of conditions; (6) presence of hypertension defined as systolic blood pressure values over 140 and/or diastolic over 90 mmHg [26]; (7) body mass index (BMI), recorded by a trained nurse, and (8) depressive symptoms assessed through the Center for Epidemiological Studies Depression (CES-D) [27]; and (9) the use of non-steroidal anti-inflammatory drugs (NSAIDs).

### Statistical analyses

For continuous variables, normal distributions were tested using the Kolmogorov-Smirnov test. The data are reported as means and standard deviations (SD) for continuous measures, and percentages for all categorical variables by mitochondrial haplogroups. For continuous variables, differences between the means of the covariates by mitochondrial haplogroups were calculated using an Analysis of Variance (ANOVA); chi-square test was applied for categorical variables. Levene’s test was used to test the homoscedasticity of variances and, if its assumption was violated, then Welch’s ANOVA was used. Post-hoc analyses were applied to compare data. All the p-values were reported taking the haplotype HV (the most common) as reference.

The strength of the association of mitochondrial haplogroups and incident CVD was assessed through a Cox’s regression analysis. Time to event was calculated as time to CVD or to the last observation made not including the people with CVD at baseline. Dead participants were censored at the time of death. Incidence rate was reported as the number of people having CVD during follow-up per 1,000 persons-year, with the 95% confidence intervals. Factors significantly associated with CVD at follow-up in the univariate analysis (taking a p-value<0.05 as statistically significant) were included in the model. Multi-collinearity among covariates was assessed through variance inflation factor, with a cut-off of 2 as a reason of exclusion. No variable was excluded due to this reason. Data of Cox’s regression analysis were reported as hazard ratios (HRs) with 95% confidence intervals (CIs).

All analyses were performed using the SPSS 17.0 for Windows (SPSS Inc., Chicago, Illinois). All statistical tests were two-tailed and statistical significance was assumed for a p-value <0.05.

## Results

### Study participants

At baseline, among 4,796 potentially eligible individuals, 313 already had a CVD, 866 subjects did not have a mitochondrial DNA assessment and the other 126 did not have this assessment for technical problems. Finally, 203 participants were lost at follow-up. Thus, 3,288 participants were enrolled in the current study.

### Baseline analyses

The 3,288 participants included aged 61.3±9.2 (range: 45–79) years, with slightly more women (= 56.8%). All the participants included were Caucasians.

The baseline characteristics of the participants by mitochondrial haplogroups are reported in Table 1. The HV group was the most frequent haplotype (n = 1,497) and was used as the reference in all the elaborations. Compared to the HV haplogroup, no differences emerged in terms of age, sex, BMI, NSAIDs use, hypertension or diabetes across the other groups. On the contrary, the participants in the superHV/others haplogroup (n = 184) reported a significantly lower PASE score (p = 0.03) indicating a lower physical activity level and a lower education level (p = 0.02). Finally, the IWX haplogroup (n = 141) reported a higher prevalence of people having a low yearly income (p = 0.001) and a significant higher number of comorbidities (p = 0.04), when compared to the HV haplogroup.

**Table 1:** Baseline characteristics by mitochondrial haplogroups.

*Variable*	HV (n = 1497)	JT (n = 659)	UK (n = 807)	IWX (n = 141)	SuperHV/ Others (n = 184)
**Age (years)**	61.1 (9.2)	61.2 (9.4)	61.7 (8.9)	60.9 (9.6)	61.3 (9.4)
**Females (%)**	58.2	56.9	54.6	53.2	57.1
**BMI (Kg/m**^**2**^**)**	28.1 (4.6)	28.1 (4.7)	28.0 (4.7)	28.2 (4.7)	28.0 (4.3)
**PASE (points)**	166.5 (82.2)	163.9 (78.0)	165.7 (80.2)	174.0 (78.3)	146.4 (82.2) *
**Smoking (%)**	45.1	47.2	45.2	46.8	45.9
**Degree (%)**	33.5	32.8	35.9	37.9	28.8 *
**Yearly income (<50,000 $)**	29.9	29.6	30.1	41.1 **	35.9
***Medical conditions***					
**NSAIDs use (%)**	36.7	36.7	41.1	37.9	36.6
**Hypertension (%)**	18.6	20.5	16.0	19.1	16.3
**Diabetes (%)**	5.2	4.0	4.4	8.0	6.1
**Charlson co-morbidity score**	0.2 (0.7)	0.2 (0.6)	0.2 (0.6)	0.4 (0.9) *	0.4 (1.0)
**CES-D (points)**	6.1 (6.6)	5.8 (6.1)	5.8 (6.1)	6.3 (6.7)	6.3 (6.7)

Notes:

In all the comparisons, the group HV was taken as reference.

**: p<0.001

*: p<0.05.

Numbers are mean values (and standard deviations) or percentages, as appropriate.

Abbreviations: BMI: body mass index; CES-D: Center for Epidemiological Studies Depression; NSAIDs: non-steroidal anti-inflammatory drugs; PASE: physical activity scale for the elderly.

### Association between mitochondrial haplogroups and CVD

After a median period of 8 years, 322 individuals (9.8% of baseline population) developed a CVD.

**Fig 1:**
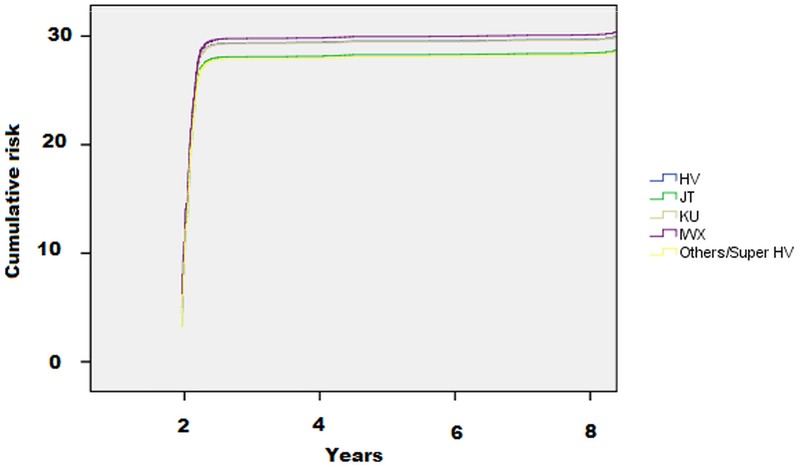
Risk of cardiovascular disease by mitochondrial haplogroups. The data are reported as hazard ratios.

As shown in Table 2 and in Fig 1, the incidence of CVD was lower in people with the haplotype JT (11; 95%CI: 8–14 per 1,000 persons-year) and Others/superHV haplogroups (10; 95%CI: 6–17 per 1,000 persons-year) compared to the reference group, HV (14; 95%CI: 12–16 per 1,000 persons-year).

**Table 2:** Association between mitochondrial haplogroups and incident cardiovascular disease.

	Number of people at baseline	Incidence of CVD (95% CI) per 1,000 persons-year	Basic adjusted HR^a^ (95%CI)	P value	Fully adjusted HR^b^(95%CI)	P value
**HV**	1497	14 (12–16)	1 [reference]		1 [reference]	
**JT**	659	11 (8–14)	0.75 (0.54–0.98)	0.03	0.75 (0.54–0.96)	0.03
**UK**	807	15 (12–19)	0.98 (0.75–1.28)	0.89	0.99 (0.76–1.30)	0.94
**IWX**	141	17 (10–26)	1.18 (0.71–1.94)	0.53	1.11 (0.67–1.83)	0.70
**Others/ SuperHV**	184	10 (6–17)	0.75 (0.43–1.29)	0.30	0.72 (0.41–1.25)	0.24

Notes:

^**a**^ Basic adjusted model includes age (as continuous) and sex.

^**b**^ Fully-adjusted model included as covariates: body mass index (as continuous); education (degree vs. others); smoking habits (current and previous vs. others); yearly income (categorized as ≥ or < 50,000$ and missing data); presence of hypertension (yes vs. no); Charlson co-morbidity index (as continuous); Physical Activity Scale for Elderly score (as continuous); Center for Epidemiological Studies Depression; use of non-steroidal anti-inflammatory drugs (yes vs. no).

Abbreviations: CI: confidence intervals; HR: hazard ratio.

Using a Cox’s regression analysis adjusted for eleven potential confounders and taking people with the HV haplotype as reference, the haplogroups JT carried a significant lower risk of incident CVD of 25% (HR = 0.75; 95%CI: 0.54–0.96; p = 0.03) (Table 2).

In a sensitivity analysis, we separated the haplogroup J from the T. After adjusting for the same potential confounders, the haplogroup J was protective for the onset of CVD (HR = 0.71; 95%CI: 0.46–0.95; p = 0.02) in 306 participants, whilst the haplogroup T was not associated with any significant reduction in CVD incidence (HR = 0.75; 95%CI: 0.50–1.13; p = 0.18) in 353 individuals (other details not shown).

## Discussion

In this paper, in a cohort of people having knee OA or at high risk for this condition, over a follow-up period of 8 years, we found that people who had an JT haplogroup carried a significantly lower risk of CVD of 25%, compared to the most frequent haplotypes (HV). This finding remained significant after multivariable adjustment for several potential confounders assessed at baseline.

It is noteworthy that at baseline, participants with the JT haplogroup did not have any significant difference in potential CVD risk factors, including obesity, presence of hypertension or diabetes. However, after 8 years of follow-up, the JT haplogroup (and particularly the J) carried a consistent reduction in CVD incidence, suggesting an important role of these haplotypes in CVD prevention. The haplogroup J seems to be a potentially protective factor for other disease, such as knee osteoarthritis [20, 28]. Similarly, two case-control studies reported that people having J haplogroup reported a significantly lower prevalence of CVD compared to the other mitochondrial haplogroups [15, 16].

Several reasons could explain our findings. It is known that haplogroup J typically has lower oxygen consumption than other mitochondrial haplogroups, with a consequent lower efficiency in the electronic respiratory chain and low reactive oxygen species production [29, 30]. Thus, participants with a mitochondrial haplogroup J undergo less mitochondrial oxidative damage than other haplogroups, leading to less consumption of oxygen by the heart [31]. Since oxidative stress and inflammation [32] are two key pathways in the development of atherosclerosis, we can argue that this haplotype may carry a lower risk of CVD in our population. Moreover, some mitochondrial haplogroups have also been associated with longevity. Again, the haplogroup J was overrepresented in Finnish centenarians [33], suggesting that this haplogroup confers an important protection for several medical conditions in human beings.

However, we should note that a large prospective study did not find any association between J haplogroup and the risk of ischemic CVD or mortality [18]. Contrary to the findings of case-control studies [15, 16], in this prospective study mitochondrial haplogroups were not associated with any significant reduction in CVD incidence. We can hypothesize that adjustment for multiple covariates and the use of the Bonferroni’s correction in the analyses of this cohort study nullified the association between mitochondrial haplogroups and CVD, in contrast to case-control which did not use these approaches [18]. In this regard, larger cohorts are needed to understand the possible associations between mtDNA haplogroups and medical conditions [34]. Other studies reported that other haplogroups confer a protection for ischemic transient or definitive cerebrovascular diseases, but not against myocardial infarction [35]. Thus, future longitudinal research is needed to fully understand the role of the mitochondrial haplogroups in the development of CVD.

The findings of our study should be interpreted within its limitations. First, the presence of CVD was assessed through self-reported information and this could have biased our results towards the null due to misclassifcation, although one would anticipate that this would have affected the results similarly across mitochondrial haplogroups. Second, we did not assess the role of medications for the prevention/treatment of CVD. Third, data were only available of mitochondrial haplogroups among Caucasians and the results do not extend beyond this ethnicity. On the contrary, increasing research is showing the importance of mtDNA in predicting chronic diseases in Africans [36, 37] and consequently, future research is needed to understand the role of mtDNA in these ethnicities. Finally, the OAI included only people with or at high risk of knee osteoarthritis and so it could be not fully representative of the general population. Thus, future longitudinal research in general population is required to address these inconsistencies and inadequacies. Finally, we did not use P-values lower than 0.05 to denote statistical significance as methods such as the Bonferroni correction have been criticized for reasons such as high risk for type II errors, and the lack of consensus on how many comparisons warrants this correction [38]. However, it is worth noting that with the application of the strict Bonferroni correction, our main findings will become non-significant. Among the strengths of our work, we can emphasize the study design (longitudinal vs. the other case-control studies), the large cohort included at baseline and the long-follow-up period. These last factors seem to be essential since, very large cohorts are required to detect significant associations with human medical diseases.[34].

In conclusion, in our study, we found that mitochondrial haplogroups JT may carry a significant lower risk of CVD compared to the most frequent haplogroups, HV. However, it is worth noting that this result was not based on a high level of statistical significance and that with the use of lower P-values to denote statistical significance (e.g., Bonferroni correction), this result is no longer significant. This indicates that our study finding is weak in terms of statistical credibility and that other research with larger populations is needed. Since sequencing of the complete mtDNA genome is now readily available, in the coming era of personalized medicine, these types of genetic discoveries should be integrated into clinical practice for tailored therapeutic intervention strategies. [39].

## Supporting information

S1 Table Primer sequences for PCR multiplex, PCR-RFLP, and SBE reactions. (DOCX)
