# Supplementary material for: Mitochondrial genetic haplogroups and cardiovascular diseases: Data from the Osteoarthritis Initiative
Source: PLoS One. 2019 Mar 28;14(3):e0213656. doi: 10.1371/journal.pone.0213656 (PMC6438497; doi:10.1371/journal.pone.0213656)
Supplement: S1 Table — (DOCX) [file pone.0213656.s001.docx]

**Supplemental Table 1. Primer sequences for PCR multiplex, PCR-RFLP, and SBE reactions^*^**

| **Polymorphic site** | **PCR primer** | **Position** | **SNP** | **SBE primer** | **Position** |
| --- | --- | --- | --- | --- | --- |
| 7025 | 5’-CTGACTGGCATTGTATTAGCA-3’  5’-GTATACGGGTTCTTCGAATG-3’ | 6960F  7433R | T7028C | 5’-ACACGACACGTACTACGTTGTAGC-3’ | 7004F |
| 14766 | 5’-GAGAAGGCTTAGAAGAAAACCCCAC-3’  5’-GTGGGCGATTGATGAAAAGGC-3’ | 14601F  14950R | T14766C | 5’-cgatcATGAGTGGTTAATTAATTTTATTAGGGGGTTA-3’ | 14798R |
| 10394 | 5’-GGCCTATGAGTGACTACAAAAA-3’  5’-TATTCCTAGAAGTGAGATGGT-3’ | 10364F  10526R | A10398G | 5’-ataTATGAGTGACTACAAAAAGGATTAGACTGA-3’ | 10368F |
| 4577 | 5’-CCTACCACTCACCCTAGCATTAC-3’  5’-TAGGAATGCGGTAGTAGTTAG-3’ | 4185F  5120R | G4580A | 5’-(at)_7_TTTTTTACCTGAGTAGGCCTAGAAATAAACAT-3’ | 4548F |
| 12308 | 5’-CAACCCCGACATCATTACCGGGT-3’  5’-GGGTTAACGAGGGTGGTAAGG-3’ | 12106F  12413R | A12308G | 5’-(tacg)_5_aCCATTGGTCTTAGGCCCCAA-3 | 12288F |
| 4216 | 5’-CCTACCACTCACCCTAGCATTAC-3’  5’-GCGAGCTTAGCGCTGTGATGAG-3’ | 4185F  4542R | T4216C | 5’-cgCCACTCACCCTAGCATTACTTATATGA-3 | 4189F |
| 10032^†^ | 5’-CTTTGGCTTCGAAGCCGCCGCC-3’  5’-TATTCCTAGAAGTGAGATGGT-3’ | 9902F  10526R | A10029G | - | - |
| 14465 | 5’-ATGCCTCAGGATACTCCTCAATAGCCATC-3’  5’-CCGTGCGAGAATAATGATGTATGC-3’ | 14430F  14686R | T14470C | T14798C (J1): 5’-CCCCCTAATAAAATTAATTAACCACTCA-3’ | 14770F |
| 8994^†^ | 5’-TAGCCCACTTCTTACCACAAGGC-3’  5’-GTGTGAAAACGTAGGCTTG-3’ | 8900F  9172R | G8994A | G15257A (J2): 5’-(ta)5TTCAATGAATCTGAGGAGGCTACTCAGTA-3’ | 15228F |

**^*^** Forward (F) and reverse (R) orientations of the polymerase chain reaction (PCR) and the single-base extension (SBE) primer positions are indicated. Lower-case letters in SBE primers indicate nonspecific nucleotides at the 5 end. RFLP = restriction fragment length polymorphism; SNP =single-nucleotide polymorphism. ^†^ Digested PCR products appear as 3 fragments in an agarose gel, whereas undigested products appear as 2 fragments.
